# Surgical management of abdominal wall sheath and rectus abdominis muscle endometriosis: a case report and literature review

**DOI:** 10.3389/fsurg.2023.1335931

**Published:** 2024-01-11

**Authors:** Olga Triantafyllidou, Nikoletta Mili, Theodoros Kalampokas, Nikolaos Vlahos, Emmanouil Kalampokas

**Affiliations:** Second Department of Obstetrics and Gynecology, Medical School, National and Kapodistrian University of Athens, Aretaieio Hospital, Athens, Greece

**Keywords:** endometriosis, scar endometriosis, abdominal wall, cesarean section, parietal repair

## Abstract

**Introduction:**

Endometriosis, defined as the presence of endometrial glands and stroma outside the uterine cavity, mainly affects the pelvic viscera and peritoneum. Endometriosis can also occur at sites of surgical incisions on the abdominal wall, mainly in women with a history of cesarean section (CS). The incidence of abdominal wall endometriosis after CS reaches 1%. Clinical suspicion, along with imaging, plays a crucial role in diagnosis. The preferred treatment involves extensive surgical excision with clear margins, ensuring a definitive diagnosis through histopathology examination.

**Case presentation:**

This case report is of a 44-year-old woman with a history of two CS procedures who developed pain and pigmentation at the incisional site one year after the last CS. Thirteen years after the surgical excision of an abdominal wall endometriosis (AWE) mass, followed by hormone therapy, she presented in our hospital with worsening pain for further management. Pelvic MRI findings were consistent with AWE. During surgery, the abdominal wall endometriosis foci were removed, and the defect in the aponeurosis was repaired using a dual-sided mesh in a tension-free procedure.

**Conclusion:**

Although AWE is a rare condition, we foresee an increase in cases because of the ever-increasing CS rates and the important association between AWE and CS. Healthcare practitioners should remain vigilant for this condition in women of reproductive age who exhibit cyclic pain, a palpable mass in the abdomen, and a background of previous uterine surgeries.

## Introduction

Endometriosis, marked by the presence of endometrial glands and stroma outside the uterus, is a prevalent gynecological disorder affecting up to 10% of women during their reproductive years (1). While it typically occurs in pelvic viscera and peritoneum, it can also, albeit rarely, affect abdominal organs, such as the kidneys, liver, pancreas, and biliary tract, as well as the diaphragm, pleura, lung, and the central nervous system (2). Some even more uncommon instances, such as nasal endometriosis, have been documented in the literature (3). Deeply infiltrating endometriosis (DIE), characterized by a solid mass situated more than 5 mm beneath the peritoneum, is often found in the pelvic region, primarily within the rectal wall and at the rectosigmoid junction. It can also occur in other organs like the bladder or ureters (4). Additionally, endometriosis may manifest at surgical incision sites on the abdominal wall following procedures such as laparotomies, laparoscopic port sites, and hernia repairs (5).

The term “abdominal wall endometriosis” (AWE) encompasses ectopic endometrial tissue located superficial to the peritoneum. The prevailing theory regarding the pathogenesis of AWE is iatrogenic direct implantation, where endometrial tissue is inadvertently introduced into the surgical wound during procedures (6). The majority of AWE cases are observed in women with a history of cesarean section (CS) (7). The incidence of AWE after CS ranges from 0.03% to 1% (7). CS rates have been steadily rising worldwide and have approximately doubled since 2000, with rates reaching 27% in Western Europe, 32% in North America, and 44% in Latin America (8). Given the strong association between AWE and CS, it is expected that the number of cases will continue to rise due to the rising rates of CS.

## Case report

A 44-year-old woman presented to our hospital with cyclical abdominal pain, primarily located near the left side of her cesarean section (CS) scar. During the physical examination, a palpable mass was identified on the left side of the CS scar, while a bimanual examination did not reveal any abnormalities within the pelvis. Her obstetric history included two previous CS procedures one 17 and the other 15 years ago. Additionally, she had undergone surgical excision of an AWE mass 13 years previously. Following her second CS, the patient developed cyclical pain and skin pigmentation at the CS scar site. Subsequently, she underwent surgical excision of an AWE lesion on the right side of the CS scar, as confirmed by the pathology report. Initially, the patient experienced relief from symptoms after the surgery. However, five years later, the symptoms recurred.

To manage the pain, the patient was prescribed 2 mg dienogest per os daily for eight years, which provided adequate symptom control. Two months ago, due to worsening pain, she received two monthly courses of 3.75 mg triptorelin intramuscularly; however, it did not alleviate her symptoms. Consequently, she sought further management at our hospital.

**Figure 1:**
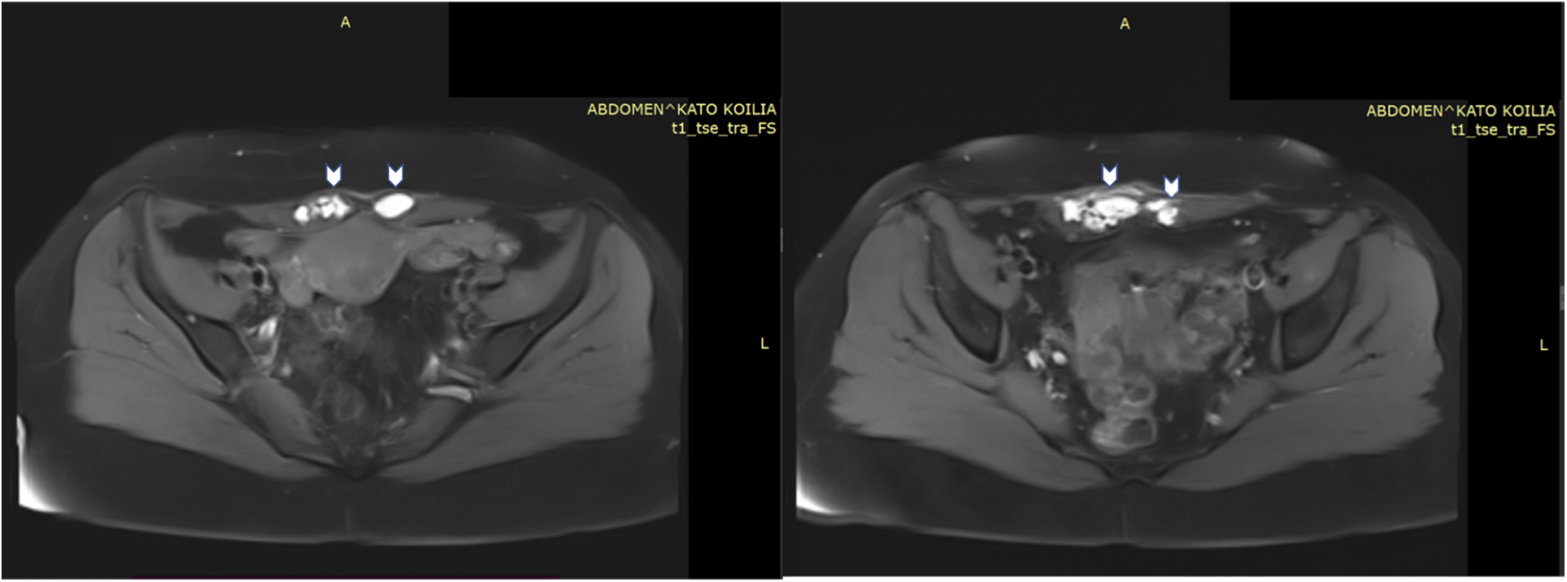
MRI images of lesions (arrows) within the right and left rectus abdominis muscles, exhibiting a hyperintense signal on T1- weighted images.

Pelvic magnetic resonance imaging (MRI) revealed multiple round and ovoid formations within the lower region of both the right and left rectus abdominis muscles, exhibiting a hyperintense signal on both the T1- and T2-weighted images. Similar lesions were also found within the subcutaneous tissue near the outer lower region of the rectus abdominis muscle, measuring 13.1 × 17.8 mm. The MRI also indicated findings consistent with adenomyosis. Signs of pelvic endometriosis were not reported in the MRI report (Figure 1). Her cancer antigen 125 (CA125) and carbohydrate antigen 19-9 (CA19-9) levels were 51.40 U/ml and 464.42 U/ml, respectively. Considering the patient's past history of AWE, typical clinical presentation, and imaging findings, the most likely diagnosis was a recurrence of AWE. Therefore, surgery for lesion excision and histological confirmation was scheduled.

**Figure 2:**
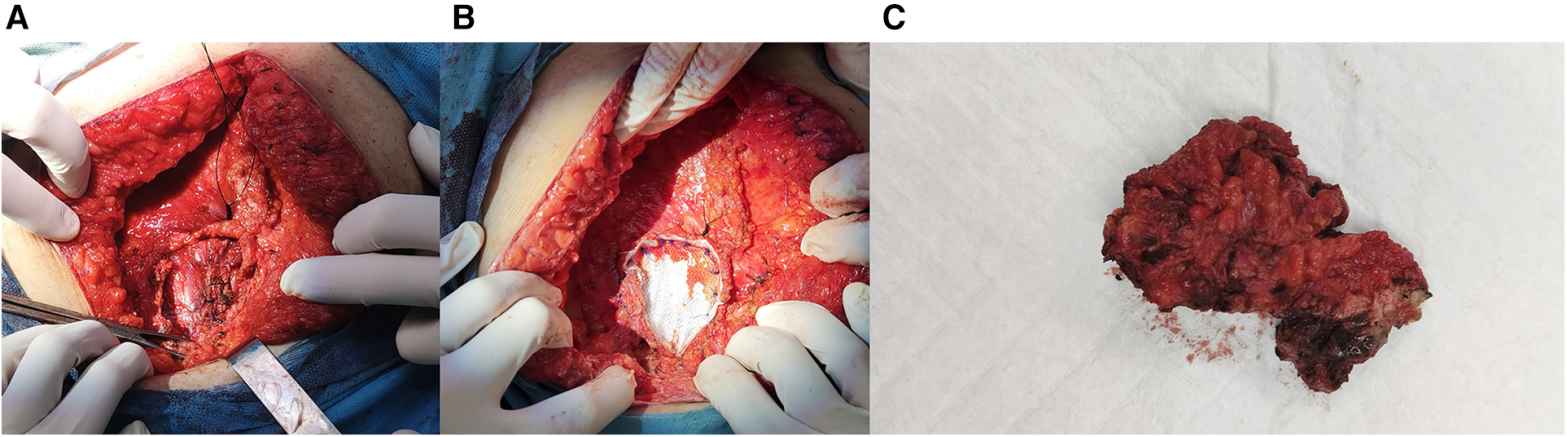
(**A**) Muscle and fascia defect measuring 7 cm × 5 cm in the abdominal wall. (**B**) Repair of the defect with mesh. (**C**) Resected AWE mass.

Laparotomy was performed, and an endometrioid lesion measuring 7 × 5 cm was found in the midline of the rectus abdominis sheath. Additionally, another lesion measuring 1.5 × 1 cm was identified on the left external oblique muscle. A chocolate-like fluid sac was found within the larger AWE lesion. Both AWE lesions were surgically removed with negative surgical margins as confirmed by the pathology report. Considering the patient's documented history of histologically confirmed AWE and the typical presentation, the risk of malignancy was deemed low. Consequently, no indication for a frozen section was present. The defects in the aponeuroses were repaired using tension-free procedures with dual-sided meshes (Figure 2).

The pathology report identified both specimens as “endometriotic cysts” noting presence of hemosiderin, histiocytes, endometrial stroma and cystic dilatation of endometrial glands with intraluminal necrosis, hemorrhage, histiocytes, nuclear debris, and inflammatory cells. The glands exhibited moderate nuclear atypia and hobnail cell appearance. Surgical margins were negative in both specimens.

The patient recovered smoothly without complications and was discharged just two days after surgery. She has since found relief from the cyclic pain, achieving the desired outcome. Even though the patient was disappointed by the recurrence of AWE and the need for a second surgery, she found satisfaction in the easy and uncomplicated recovery, allowing for a swift return to her everyday routine. With symptoms relieved after recovery, she feels content with the decision to opt for surgical management following the failure of medical interventions.

**Figure 3:**
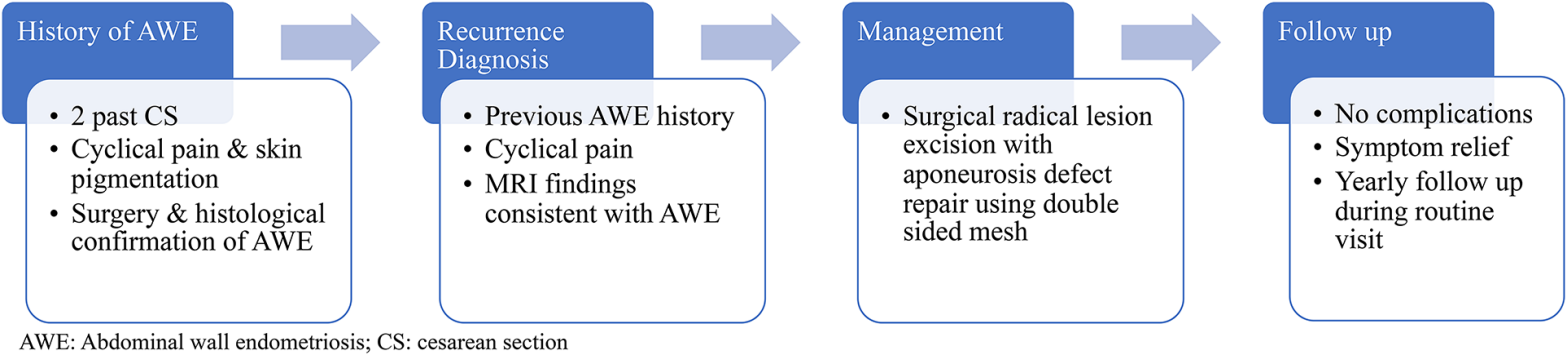
Episode of care timeline.

Given that the diagnosis of AWE recurrence is primarily established clinically based on symptoms, the follow-up plan now includes interview and physical examination during her annual routine gynecological care visits (Figure 3).

## Discussion

A typical presentation of AWE includes the classic triad of symptoms: a palpable mass, often accompanied by cyclical pain, and a history of a previous surgical incision (9). Superficially located endometriomas may exhibit cyclic external bleeding or ulceration, indicating the presence of a prolonged disease or a fistula tract (10, 11). In a comprehensive review of 445 cases, 96% of the patients presented with a palpable mass, and 87% experienced pain, with 57% reporting cyclical pain. Among patients with a previous CS, pain near the CS incision site was a common complaint. Additional symptoms, such as dysmenorrhea, pelvic pain, dyspareunia (painful intercourse), and bowel or bladder disturbances, were also observed in many patients (7).

The incidence of AWE following surgery for endometriosis and CS has been reported to range from 0.04% to 12% and 0.03% to 1%, respectively (7, 12). In published case series, the majority of patients with AWE had a history of prior surgery, most commonly a CS (7, 10, 12, 13). Rates of concurrent pelvic endometriosis in patients with AWE vary across studies, ranging from 0% to 34.2% (14). In a review of 445 cases, Horton et al. found that 20% of patients with AWE did not have a history of prior surgery, 57% had a CS scar, 11% had a hysterectomy scar, and 13% had other surgical scars. Symptoms typically appeared around 3.6 years after the initial surgery. Only 13% of patients with AWE received prior or subsequent pelvic endometriosis treatment (7). Another study by Ecker et al. reported that 80% of the study population had a history of CS, and the interval between the original surgery and the onset of symptoms ranged from 1 month to over 10 years (12) (Table 1).

**Table 1:** Characteristics of abdominal wall endometriosis case series.

Author, year	Number of cases	Symptoms			Surgical history			Interval from the original surgery to presentation	Mean diameter (cm)	Recurrence
		Pain	Cyclic Symptoms	Mass	Cesarean section	Other	None			
Horton, (7)	445	87%	57%	96%	57%	13%	20%	3.6 years	2.7	4.3%
Bektas, (10)	40	45%	40%	100%	90%	10%	0	29.6 ± 27.5 months	4.6 ± 1.9	9.1%
Ecker, (12)	65	73.8%	N/A	63.1%	81.5%	12.3%	6.2%	7 years (IQR 4-11.5)	4.8 cm	N/A
Ramos-Mayo, (13)	29	N/A	68.9%	100%	100%	N/A	N/A	N/A	Size 13.48 cm^2^	0%
Marras, (14)	35	N/A	Pain 68.8%	25.7%	65%	13.2%	17.1	5.28 ± 3.7 years	2.38 ± 1.24	11.4%
			Bleeding 11.4%							
Foley, (15)	32	N/A	Pain 75%	54%	95%	N/A	N/A	N/A	4.62 cm	N/A

Women who have undergone a previous CS typically exhibit lesions that are primarily situated in the surgical scar. However, in patients without a history of CS, the main location for lesions is the umbilicus (12, 14). Several studies have indicated that masses tend to be more frequently found on the left margin of incision scars (12, 16, 17).

The range of potential causes for an abdominal wall mass linked to previous surgical incisions is extensive, encompassing benign conditions like hernias, excessive fibroses, suture granulomas, abscesses, and rarely, malignant conditions, such as sarcomas and metastatic diseases (11). A thorough history-taking and physical examination typically play a crucial role in accurately diagnosing AWE (11). During the physical examination, it is essential to determine if there is a fascial defect and whether the mass appears to be connected to the anterior fascia. In cases where the presentation aligns with typical symptoms, additional studies may not be necessary (18).

In situations where suspected malignancy may warrant more aggressive management, procedures like percutaneous biopsy, fine needle aspiration (FNA), and frozen section during surgery can play a role in avoiding overtreatment. However, it's important to note that in many cases, neither FNA nor tissue biopsy provides conclusive evidence of the presence of endometriosis. The traditional “triad” of endometrial glands, stroma, and hemosiderin-laden macrophages, commonly used as diagnostic criteria, is observed in only a third of surgical samples. In cases where endometrial glands are absent or only a small area of endometrial stroma is detected, immunohistochemistry has been suggested as the most useful diagnostic (2).

In cases where the lesion is particularly large, concerns arise about extensive disease involving the fascia and the potential need for mesh reconstruction, or when uncertainty surrounds the diagnosis, additional imaging studies may become necessary. These supplementary data can offer valuable insights for surgical planning, especially when abdominal wall reconstruction is anticipated (7). The sonographic pattern of AWE is characterized by a distinct hypoechoic subcutaneous nodule with irregular margins that extend to the muscularis fascia. A single vascular pedicle entering the mass at the periphery may also be observed. Often a hyperechoic ring surrounds the nodules, believed to be caused by an inflammatory reaction. In larger lesions, small cystic areas may be visible, potentially indicating recent hemorrhage and the formation of blood-filled spaces (19). On MRI, most lesions exhibit a hyperintense signal on T1- and T2-weighted images, indicating the presence of blood products within the lesions (20).

Surgical intervention is widely considered the most appropriate approach for managing AWE due to limited success with medical management in previous studies (6, 7, 12). While oral contraceptives, progesterones, and danazol may offer temporary relief from symptoms, they do not address the underlying lesion, and there is a significant probability of recurrence after discontinuing treatment (21). For AWE, the preferred treatment involves removing the lesion through a wide local surgical excision, ensuring a margin of at least 1 cm to achieve negative margins (11). In cases in which AWE infiltrates the muscular layers of the abdominal wall, it may be necessary to perform an en-bloc resection of the underlying myofascial structures. Additionally, in cases of defects larger than 3 cm, abdominal wall reconstructions with mesh repairs may be required (14, 15). Rectus muscle-confined lesions can be effectively removed through laparoscopic or robotic excision, keeping the anterior rectus fascia intact. Both laparoscopic fascial closure devices for primary tension-free closure and laparoscopic placement of a mesh can be utilized (15). The need for mesh repair is associated with the size of the lesion, the average duration of symptoms related to the painful mass, the levels of serum CA125, the degree of penetration through the fascial layer, and the extent of invasion of the rectus abdominis muscle layer (22, 23).

The recurrence of AWE is associated with positive surgical margins, with reported recurrence rates ranging from 4.3% to 17.2% in different case series (7, 15). When an AWE recurs, surgical re-excision of the affected area is the recommended treatment approach (24).

Novel nonsurgical radiologic interventions, such as ultrasound-guided high-intensity focused ultrasound (USgHIFU) and cryoablation, have demonstrated effective reduction in pain scores and lesion sizes in cases of AWE. However, further research is required to compare the rates of complication and recurrence between these noninvasive therapies and surgery (25–27).

Given that AWE is primarily iatrogenic in nature, various preventive measures have been suggested. These include exteriorization of the uterus, omitting uterine cavity swabbing, flushing, and irrigation of the abdomen and incision, removing all instruments that have come into contact with the uterus and changing gloves for parietal closure, and using a protective bag when extracting specimens during laparoscopy to prevent the seeding of port sites (6, 7, 10, 28). However, the effectiveness of these measures has not been evaluated through prospective trials.

## Conclusion

Despite the rarity of AWE, the escalating rate of CS in recent years underscores the importance of considering this uncommon form of endometriosis when evaluating painful masses in the abdominal wall. Complete wide excision with clear margins serves a dual purpose in the management of AWE, contributing to both diagnosis and treatment. Surgeons should maintain a high level of suspicion for this condition in reproductive-age women who present with cyclic pain, a palpable abdominal mass, and a history of uterine-related surgeries.

## Data Availability

The original contributions presented in the study are included in the article/Supplementary Material, further inquiries can be directed to the corresponding author.
